# Amelioration of amyloid beta (Aβ_1-40_) neurotoxicity by administration of silibinin; a behavioral and biochemical assessment

**DOI:** 10.22038/IJBMS.2023.66831.14665

**Published:** 2023

**Authors:** Tahereh Alihosseini, Monireh Azizi, Nasser Abbasi, Shahram Mohammadpour, Maryam Bagheri

**Affiliations:** 1 Department of Anatomy, School of Medicine, Ilam University of Medical Sciences, llam, Iran; 2 Biotechnology and Medicinal Plants Research Center, School of Medicine, Ilam University of Medical Sciences, Ilam, Iran; 3 Department of Physiology, School of Medicine, Ilam University of Medical Sciences, Ilam, Iran

**Keywords:** Alzheimer’s disease, Amyloid, BDNF, Oxidative stress, Silibinin, VEGF

## Abstract

**Objective(s):**

Alzheimer’s disease (AD), the most common cause of dementia, is one of the leading causes of morbidity and death in the world. Currently, treatment mostly used to slow down the disease progression. Herbal remedies are considered by many in the community as a natural and safe treatment with fewer side effects. Silibinin, the active ingredient of *Silybum marionum*, has anti-oxidant, neurotrophic and neuroprotective characteristics. Therefore, here, the effect of different doses of Silibinin extract on oxidative stress and expression of neurotrophic factors was investigated.

**Materials and Methods:**

Forty eight male Wistar rats were randomly divided into sham, lesion; Aβ_1-40_ injection, lesion-treatment; Aβ_1-40_ injection followed by different doses of silibinin (50, 100, 200 mg / kg) through gavage and lesion-vehicle group; Aβ_1-40_ injection + vehicle of silibinin. Morris water Maze (MWM) was done 28 days after the last treatment. Hippocampal tissue was removed for biochemical analysis. Production of nitric oxide (NO) and reactive oxygen species (ROS), expression of BDNF/VEGF and cell viability were measured using Griess, fluorimetry, Western blotting and MTT techniques.

**Results:**

Different concentrations of silibinin improved behavioral performance in animals. Higher doses of Silibinin could improve memory and learning function through MWM. Also, increasing the concentration of silibinin resulted in decreased ROS and NO production in a dose-dependent manner.

**Conclusion:**

Consequently, silibinin may act as a potential candidate for alleviating symptoms of AD.

## Introduction

Neurodegenerative disorders are destructive diseases that cause social and economic damage in society (1). Today, nearly 44 million people worldwide have Alzheimer’s disease (AD). It is estimated that the prevalence of AD and related dementia diseases will reach over 130 million by 2050, which could impose very worrying costs and burdens on communities. Every five years, the prevalence of AD almost doubles with age (2). AD is characterized by degenerative brain changes that impair learning and memory (2). AD as a neurodegenerative disease is irreversible and gradually destroys memory and cognitive skills. The disease is associated with the formation of insoluble protein accumulations and loss of synapses and neuronal death (3). Abnormal accumulation of amyloid beta protein occurs outside nerve cells and they formed tau protein inside. The mechanisms that cause AD are not yet well understood. Some researchers have mentioned two causes of oxidative stress and mitotic changes as the main causes of AD (4). Oxidative stress results from an imbalance between the formation and degradation of pro-oxidants and the reduction of cellular anti-oxidant defense mechanisms, and may lead to increased cell damage and apoptosis, resulting in reduced memory (5). Free radical scavengers of reactive nitrogen species (RNS) and reactive oxygen species (ROS) damage cells by oxidizing membrane proteins and lipids, as well as DNA (6). Various show that oxidative stress plays a key role in the pathophysiology of AD and other neurodegenerative diseases, including Parkinson’s disease, Huntington’s disease and amyotrophic lateral sclerosis (ALS) (7, 8). Lipid peroxidation and subsequent production of free radicals have been reported as a factor in various diseases, including AD (9). Neurotrophins are proteins with valuable role in protection and formation of neurons. Brain-derived neurotrophic factor (BDNF), as one of the most active neurotrophin, binds to tyrosine kinase (TrKβ) receptors, triggers intracellular cascades and ultimately produces and differentiates new neurons. It was shown that the highest activity of BDNF factor is in the hippocampus and cortical part of the brain. 

Currently, the use of medicinal plants because of fewer side effects have attracted the attention of many researchers. 

*Silybum marianum* is a hairless biennial plant with thick roots. Dried seed extract of this plant contains 1 to 4% silymarin. It contains flavonoids such as silybin and its stereoisomers (isocilibin, silycristine, isosilylcristine and silydianine) (10). Silibinin is the major (70-80%) and most active compound of silymarin (11) and is said to have neuroprotective, anti-oxidant, anti-cancer and anti-inflammatory effects (12). Previous studies have shown that silibinin can induce neuroprotective effect through improving oxidative stress and inflammatory cascade in brain injury, cerebral ischemia, diabetes, and memory impairment induced by amyloid peptide β_25-35_ neurotoxicity (13, 14) and would also improve behavioral disorders following AD (15). Henceforth, the effect of different doses of silibinin extract on oxidative stress and expression of neurotrophic factors BDNF and VEGF was investigated in AD rat model. 

## Materials and Methods

***Animal***

Forty eight male Wistar rats weighing 230-250 g were used and randomly divided into six groups; Sham group with stereotaxic injection of normal saline (4 µl; n = 8), lesion group with stereotaxic injection of Aβ_1-40 _ (4 µl; n = 8), lesion-vehicle group with stereotaxic injection of Aβ_1-42 _ that daily gavaged by 1ml of hydroalcoholic solvent four weeks (n = 8), and finally the treatment groups receiving 50, 100 and 200 mg /kg silibinin (n = 8) after Aβ_1-40 _ injection; the animals received 1ml of the desired concentration of silibinin daily for four weeks. The current protocol was performed after receiving ethical approve by the ethic committee of the Ilam University of Medical Sciences numbered IR.MEDILAM.REC.1398.036.

***Surgery***

After anesthesia with ketamine/xylazine (40/10 mg / kg), the animal was first shaved and placed in a stereotaxic device. The scalp was then removed and disinfected. Based on Paxinos Watson Atlas (16) the coordinates of hippocampal injection site (AP = -3.5, ML = ± 2, DV = 2.8) were marked. Four μl of diluted Aβ_1-40_ was injected bilaterally into the hippocampus using Hamilton syringe. The injection was done slowly through 4 min, the needle was gently removed within five minutes after injection. The animal was returned to the cage after full recovery.

***Treatment preparation***

*S. marianum* aerial parts were collected from Ilam County nature; Iran. The originality of this plant was proved by the voucher specimens (No 485) by the help of the faculty of agriculture, Ilam University, Ilam, Iran. To extract the flavonolignans, 50 g powder of dried seed was placed in a Soxhlet extractor (HM6-500, UK) containing 600 ml acetonitrile at 90 °C for 3 hr. After ethanol evaporation at 50 °C, the resulting extract was kept in a 40 °C oven for 3 days to dry**.**

We have done some *in-vivo* pharmacologic trial studies to detect the suitable and safe treatment concentration based on LD50% (lethal dose). Silibinin at concentration of 50, 100, 200, 400, 600, 800, 1000, 1200, 1400 and 1600 mg/kg was investigated in animals. Dose of 50, 100 and 200 mg/kg silibinin induced no death and considered the safe dose for our experiment. 

***Behavioral tests***

*Morris water maze (MWM) test*

The device consists of a 140 cm in diameter by 80 cm high open circular pool with a hidden platform in the first quarter of the water. The test was conducted in low light, and various colored signs were posted on the walls. The main test (Probe) was carried out on the fifth day. Each rat was placed in a pool four times per day for four trial days. Each rat could swim for one minute to find the platform. The time required to find the platform; the distance traveled and the speed of swimming were recorded on each day by a camera connected to a computer. 

*Perfusion/tissue homogenization*

At the end of the behavioral test, all rats were anesthetized deeply with a high dose of ketamine. The skull was immediately cleaned**;** the brain removed and placed in a cold isotonic solution. The hippocampus was then rapidly isolated and kept at -80 °C until further study. 4 animals/ each group were undergone biochemical study.

*MTT test*

MTT test was used to measure cell viability and *the* cytotoxic effect. The method was performed using tetrazolium (di methylthiazol diphenyltetrazolium bromide) which is absorbed by the cell through endocytosis**, **and reduced to insoluble purple formazan by mitochondrial and cytosolic enzymes. The rate of formazan formation is directly related to the rate of cell growth and survival, meaning that the higher the amount of purple dye produced, the higher the number of viable cells. 

MTT test was used to evaluate the effect of solvent toxicity and different concentrations of silibinin on the growth of neurons in hippocampal tissue. First, the tissues were kept in a room temperature 30 min, then 1ml of MDEM culture medium and 50 μl of MTT (yellow) dye was added to tissue and incubated for three hours; (37 °C, 5% CO_2_). Then, 1ml of DMSO was added to stop the color performance, and finally, the optical absorption reading of the samples was performed with an ELISA reader (Model: ELX Company: Biotek USA) at 570 nm. Each experiment was repeated three times. 

*Biochemistry*

*ROS measurement*

The impact of solvent and various concentrations of silibinin extract on ROS generation in hippocampus neurons was examined using the fluorometric technique. Before adding 1 ml of culture medium (DMEM) to each well, hippocampal tissue was homogenized. Thereafter, 1 ml of 10 nm DCF dye was applied, and each well was incubated for 37 min at 37 °C with 5% CO_2_.The samples were then read with a fluorometric device (Model: FLX800 Company: Biotek, USA) at 520 nm. Each experiment was repeated three times.

*NO measurement*

Hippocampal tissue thawed at room temperature, 1 ml of MDEM culture medium was added, crushed and homogenized with an ultrasonic device (Ultrasonic Homogenizer; UP100H, Hielscher, Germany). Then 50 μl of homogeneous tissue was transferred to each well, 50 μl of sulfanilamide solution (decolorating) was added**,** and placed in a dark place for 10 min according to the instructions. Then after, NED solution (N- (1-naphthyl) ethylenediamine) was added to stop the production of NO, and finally, reading was performed with an ELISA reader (model: ELX, company: Biotek, USA) at 520- 550; each experiment was repeated three times.

***Western blotting***

The Western blotting technique was used to evaluate the effects of different concentrations of silibinin extract (50, 100 and 200 mg/Kg) on ​​the expression of neuroprotective proteins BDNF and VEGF in hippocampal tissue samples. 1 μl of culture medium was added to homogenized tissue, 300 µg of lysing buffer solution was added and kept at -80 °C. The suspension was centrifuged at 12500 rpm for 20 min. End solution was stored at -80 °C until used. Micro BCA kit (Korea Intron Company) was used to measure the total concentration of protein in the cells. For gel electrophoresis, 7 µl of protein from each sample was placed on 5.7% polyacrylamide gel and separated by electrophoresis. The proteins were transferred to a polyvinyl difluoride (PVDF) membrane and blocked for an hour at room temperature using 5% high-fat milk, 0.1% Tween 20, and a Tris saline buffer. The membranes were then incubated with primary antibody and secondary IgG conjugate antibody; Amersham pharmacia biotech Inc, Piscataway, NJ, USA (ECL) kit was used to detect the proteins. The results were measured using imaging software (Bio-rad. USA) Gel-pro analyzer. Primary antibodies were BDNF (SAB 4300702,1: 750, sigma) and VEGF (1:1000) and the secondary antibody was anti-rabbit IgG (A9169, Sigma; 1: 160000).

***Neuronal counting***

To count the number of neurons in the hippocampus of animals in different groups, they were deeply anesthetized by a high dose of ketamine. Normal saline 0.9% and Paraformaldehyde as washing and fixative solution were perfused transcardially. Brains were removed after perfusion and kept in fixative solution for a week. Paraffine embedded brains were sectioned (10 µm)**. **Three to five sections were placed on each slide and examined Nissl staining protocol. 4 animals per each group were randomly selected for Nissl staining. The number of neurons in CA1, CA3 and dentate gyrus (DG) were counted in at least three sections in each animal/ group. 

## Results

***Morris water maze***

MWM was used to evaluate memory and spatial learning. During the testing phase, the distance traveled to find the hidden platform, the time elapsed, the number of times entering into the quarter containing the hidden platform, the average speed of finding the hidden platform, and the total distance traveled to find the hidden platform were evaluated.

***The distance traveled in the first quarter to find the hidden platform***

**Figure 1:**
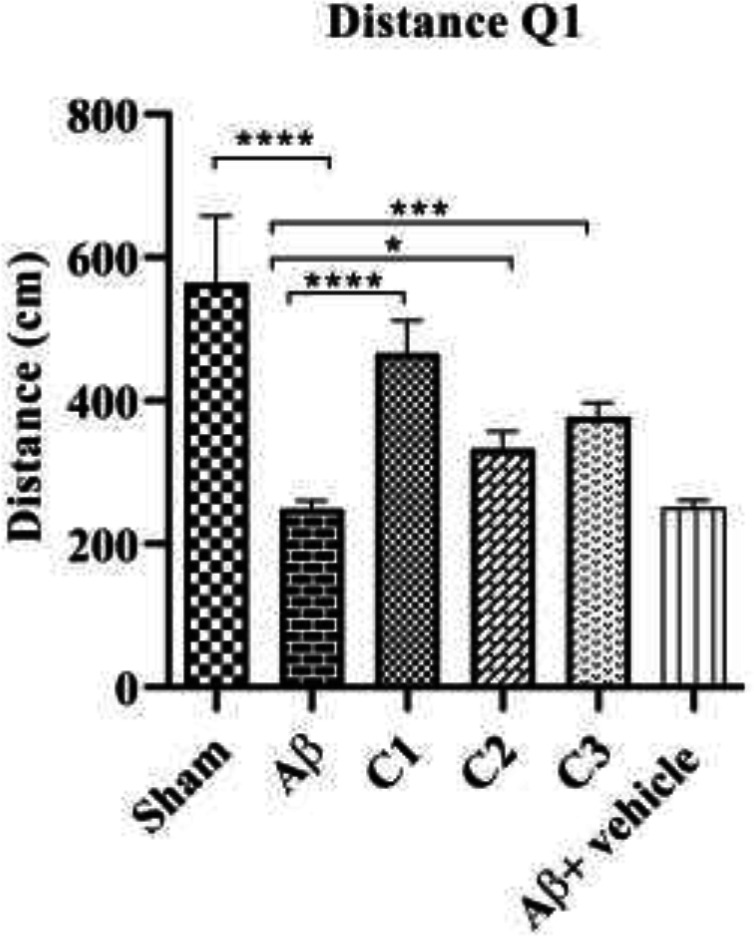
The mean distance traveled in the 1^st^ quarter of Morris water maze (MWM) in the sham, lesion (Aβ), low (C1), moderate (C2), and high (C3) concentrations of AD-treated animals, and Vehicle group (Aβ + vehicle):

As shown in Figure 1, the mean distance traveled in the first quarter of the pool was 569.5 ± 92.8 cm in the sham and was 249.0 ± 45.7 cm in the lesions which was significantly differed (*P*≤0 0.0001). On the other hand, the mean distance traveled by the treated groups with different treatment concentrations (low, medium and high dose) was 466.1 ±45.7, 333.3 ±23.5 and 377.5 ±19.58 respectively, that showed significant increase compared with the lesion group (*P*≥0.0001), (*P*= 0.01992) and (*P*=0.0006) respectively. 

***The time spent in the 1*** ^st^ ***quarter to find the hidden platform***

**Figure 2:**
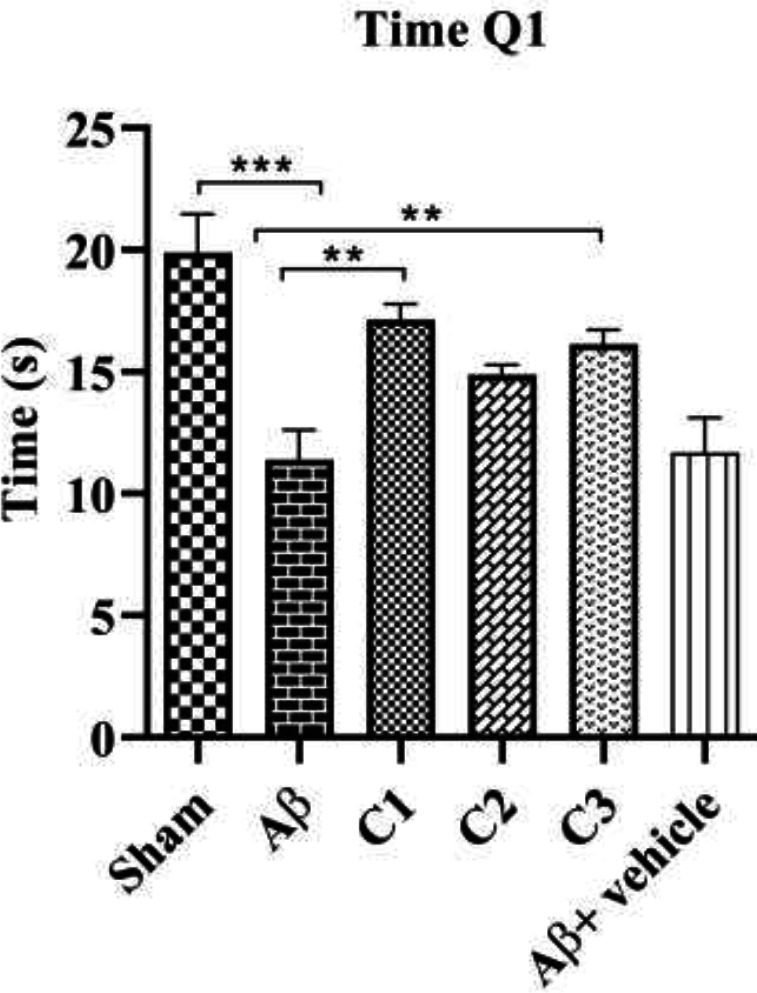
Mean time spent in Morris water maze (MWM) in the control, lesion (Aβ), lesion treated with low (C_1_), moderate (C_2_) and high (C_3_) concentration and lesion-vehicle group (Aβ + vehicle):

Figure 2 shows the time elapsed for animals in different groups to find the hidden platform. According to the results, the mean time spent in the MWM by animals was (19.88 ± 1.5) sec. in the sham group and (11.38 ±1.2) sec. in the lesion group with a significant difference (*P* = 0.0007). On the other hand, the mean time spent to find the hidden platform in the first quarter in the treated animals that received different concentrations of treatment was 17.13 ± 0.6, 14.88 ± 0.4 and 16.13±0.5 sec. respectively. Noticeably, only the treated rats with the low concentration of medication (50 mg/Kg) showed a significant increase compared to the lesioned animals (*P*≤0.001). Remarkably, there was no significant difference between lesion and lesion-vehicle groups. 

***Number of times entering the 1*** ^st^ ***quarter***

**Figure 3:**
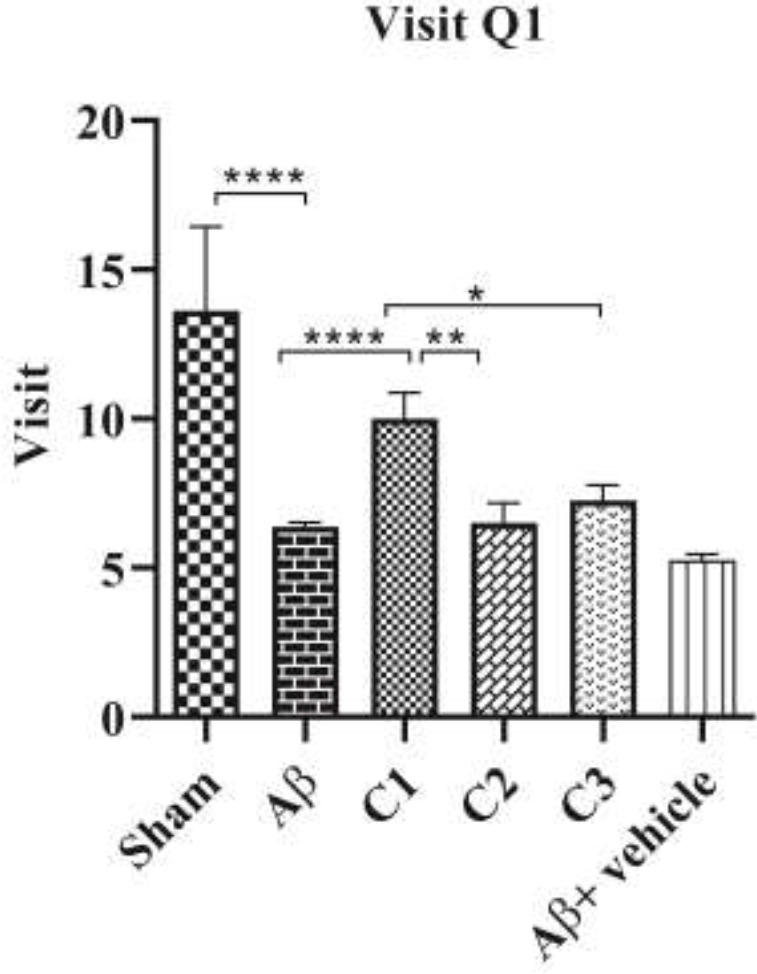
The average number of times that animals entering a quarter of Morris water maze (MWM) in the sham group, lesion (Aβ), lesion treated with low (C_1_), moderate (C_2_) and high concentration (C_3_), and lesion-vehicle group (Aβ + vehicle)

The number of times entering into the quarter containing the hidden platform is shown in Figure 3. The mean number of visits by the animals in the sham group was (13.63 ± 2.8) and in the lesion group was (6.37 ±0.14) with a significant difference (*P*≤0.0001). Furthermore, the mean number of visiting the relevant quadrant was not significant in the lesion-vehicle group compared to the lesion group. The average number of visiting in the lesioned animals receiving low concentration treatment; 50 mg/ Kg was (10 ±0.87) which were statistically increased compared with the lesion group (*P*=0.001). No significant differences were found with medium concentration treatment;100 mg/ Kg (6 ±0.6) and high concentration treatment; 200 mg/ Kg (7.2± 0.5) in compare with the lesion group. 

***The mean speed***

**Figure 4:**
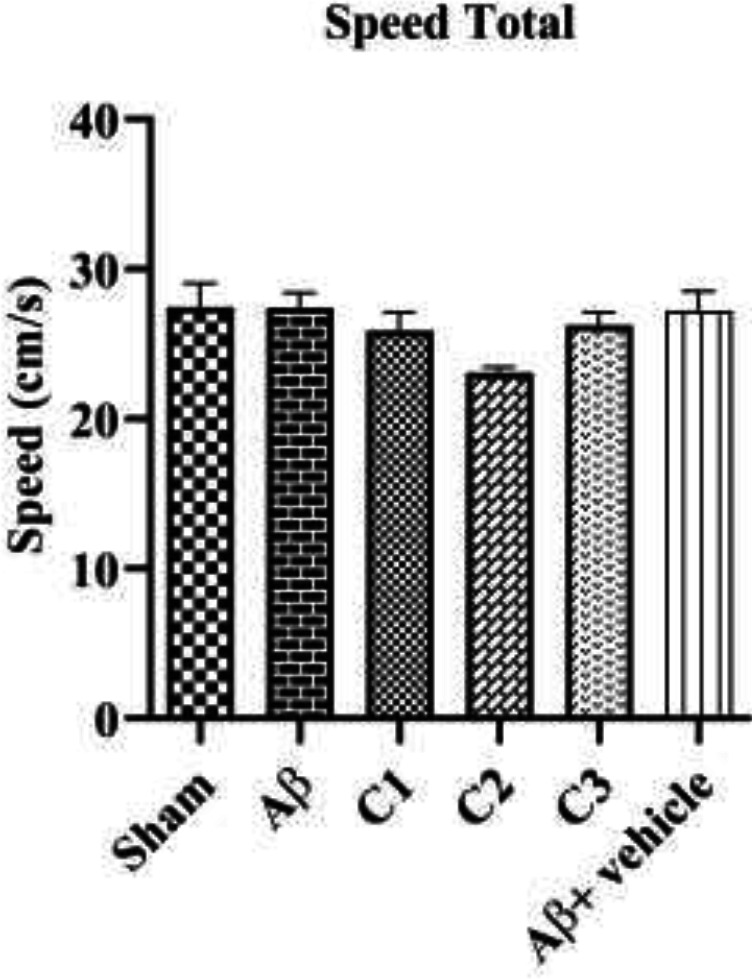
The average speed of animals in finding the hidden platform in the sham group, lesion (Aβ), Alzheimer's groups treated with low (C_1_), medium (C_2_) and high (C_3_) concentration and vehicle group (Aβ + vehicle)

To evaluate the effect of treatment or solvent on rat mobility, the mean speed of all animals in finding the hidden platform was measured. As Figure 4 shows, the mean velocity of animals was not statistically significant in all groups.

***The mean total distance traveled to find the hidden platform***

**Figure 5 a,b:**
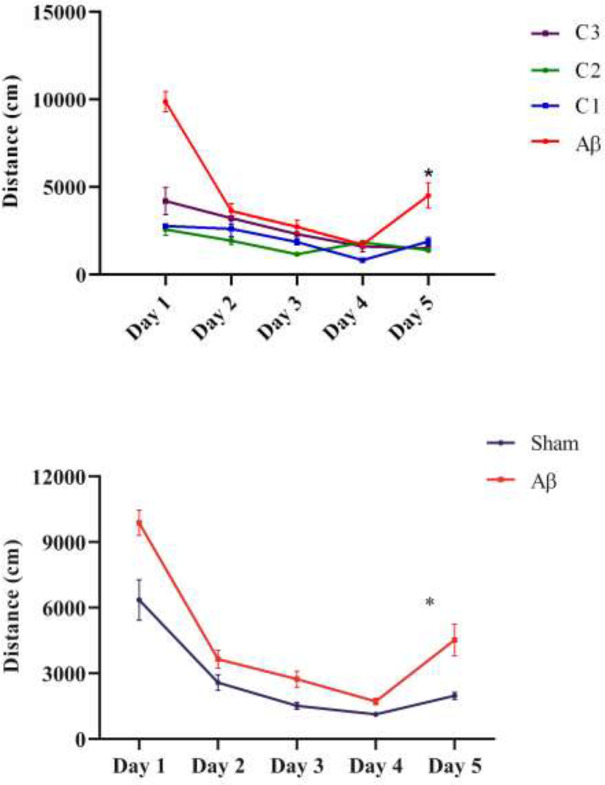
The average total distance traveled by animals to find the hidden platform in the sham and lesioned animals on different days. (C_1_ = 50 mg / kg, C_2_ = 100 mg / kg, C_3_ = 200 mg / kg)

The mean total distance traveled to find the hidden platform in the sham group was 2708 ±942.1 and in the lesion group was 4498 ± 1424 with significant difference (*P*= 0.03, Figure 5a). In addition, the mean distance traveled in the lesioned rats receiving low dose treatment was (1999 ±343.9) cm, medium dose treatment was 1789 ±243 cm and high dose treatment was 2575 ±511 cm. Totally, this factor showed a significant decrease in all groups treated with different doses compared to the untreated lesioned rats (*P*≤0.01, Figure 5b).

***Total time spent finding the hidden platform***

**Figure 6 a,b:**
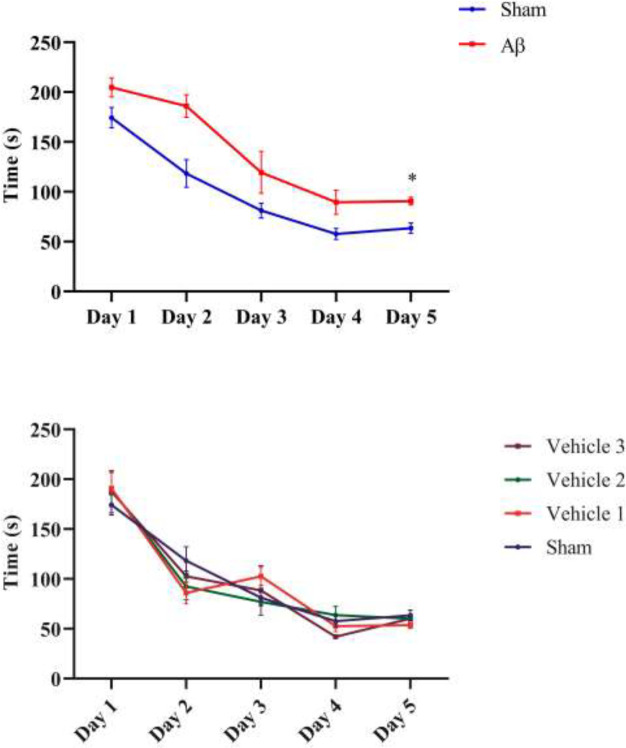
The total time spent finding hidden platform in the Control, lesioned and treated animals

The mean total time spent to find the hidden platform was 98.96 ±21.59 in the sham group and was 138.0 ±24.1 in the lesion group which was statistically increased (Figure 6a; *P*= 0.002). In addition, the mean time spent finding the hidden platform in treated animals with low dose was 21.1 ± 8.7 sec, with medium dose was 83.2 ± 13.1 sec and with high dose was 109.8 ±21.8 sec. It is noteworthy that this factor showed a significant decrease in all treated rats in compare with the untreated animals (*P*≤0.01, Figure 6b). It should be noted that the time spent by lesion-vehicle animals did not show any significant difference in compare with the untreated lesioned animals.

***MTT test***

**Figure 7:**
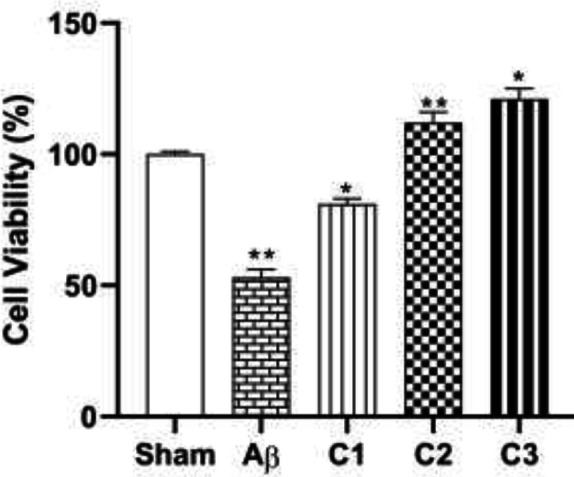
Determination of toxicity of different concentrations of Silibinin (50, 100, 200 mg / kg) on hippocampal tissue samples through MTT

This test was used to evaluate the toxicity of different concentrations (50, 100, 200 mg/ kg) of silibinin on growth inhibition and proliferation of hippocampal tissue samples. It should be noted that mitochondrial dehydrogenase enzyme can reduce MTT to formazan crystals in living cells which will develop purple color after DMSO adding. Consequently, in wells contain more living cells, more crystals will definitely form. As shown in Figure 7, in the hippocampal tissue sample of groups with different concentrations of treatment (50, 100, 200 mg/ kg), silibinin in a dose-dependent manner had a positive effect on the growth of hippocampal cells.

***ROS production***

**Figure 8:**
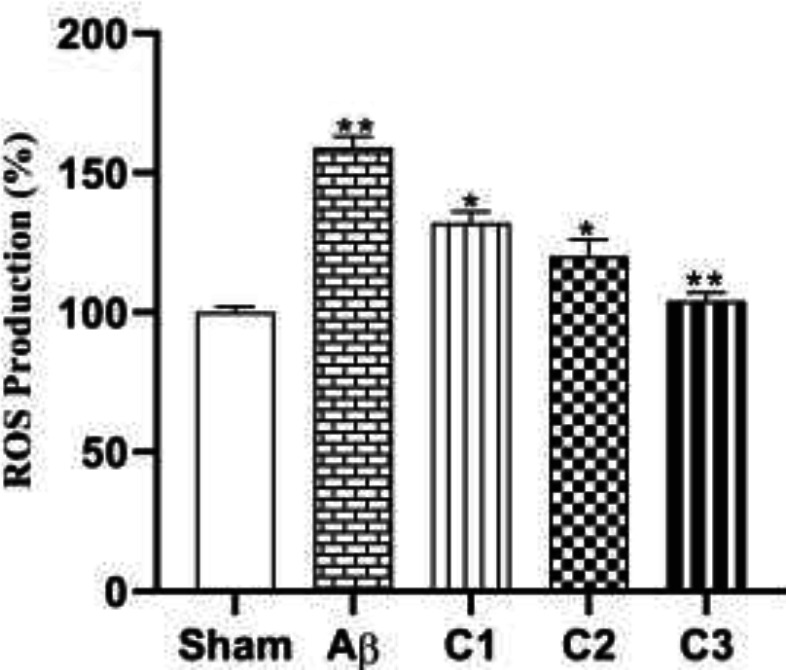
Measurement of ROS production in animals treated with different concentrations of Silibinin (C_1_=50, C_2_=100, C_3_=200 mg / kg) in compare with lesion and control groups

Different concentrations of silibinin (50, 100, 200 mg/ kg) could induce les ROS in the hippocampus of treated animals in compare with the untreated lesioned rats in a dose dependent manner (Figure 8).

***NO production***

**Figure 9:**
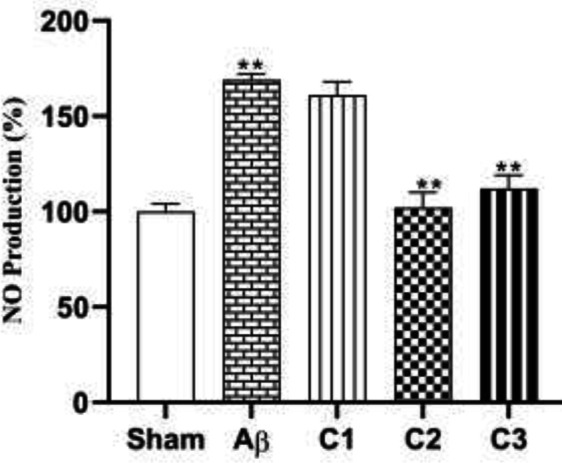
Measurement of NO production in animals treated with different concentrations of silibinin (C_1_=50, C_2_=100, C_3_=200 mg / kg) in compare with the lesion and sham groups

Different concentrations of silibinin (50, 100, 200 mg/ kg) could decrease NO production in the hippocampal tissue samples in the treatment groups compared with the lesioned rats (Figure 9).

***Western blotting***

Western blotting technique was used to evaluate the effect of different concentrations (50, 100, 200 mg/ kg) of silibinin on the expression of neuroprotective proteins BDNF and VEGF in the hippocampal samples.

**Figure 10:**
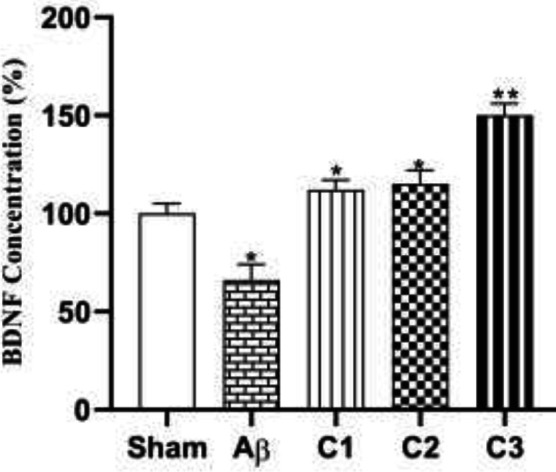
The effect of different concentrations of silibinin (50, 100, 200 mg / kg) on the expression of BDNF in the hippocampus of the Aβ-treated rats compared to Aβ injected and sham ones

**Figure 11:**
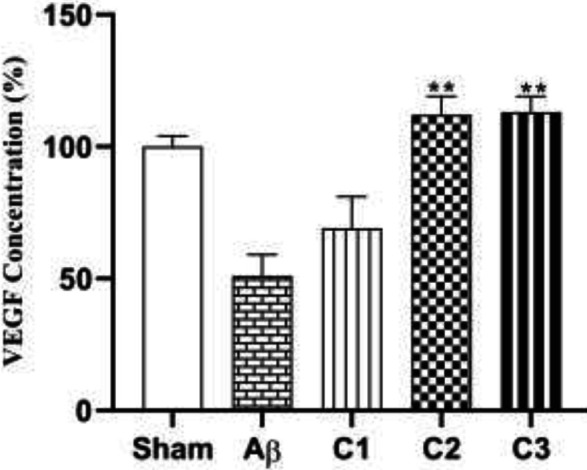
The effect of different concentrations of silibinin (50, 100, 200 mg / kg) on the expression of VEGF in the hippocampus of the Aβ-treated rats compared to Aβ injected and the sham ones. ** sham *vs*. Aβ; *P*=0.05, ** Aβ vs C_1_ and C_3_; *P*≤0.05

As shown in Figure 10, silibinin (50, 100, and 200 mg/ kg) resulted in significant increase in expression of BDNF in the hippocampus of the treated animals. Furthermore, 100 and 200 mg/kg silibinin could significantly increase VEGF concentration in hippocampal tissue of treated animals (Figure 11; *P***≤**0.05).

***Neuronal counting***

**Figure 12:**
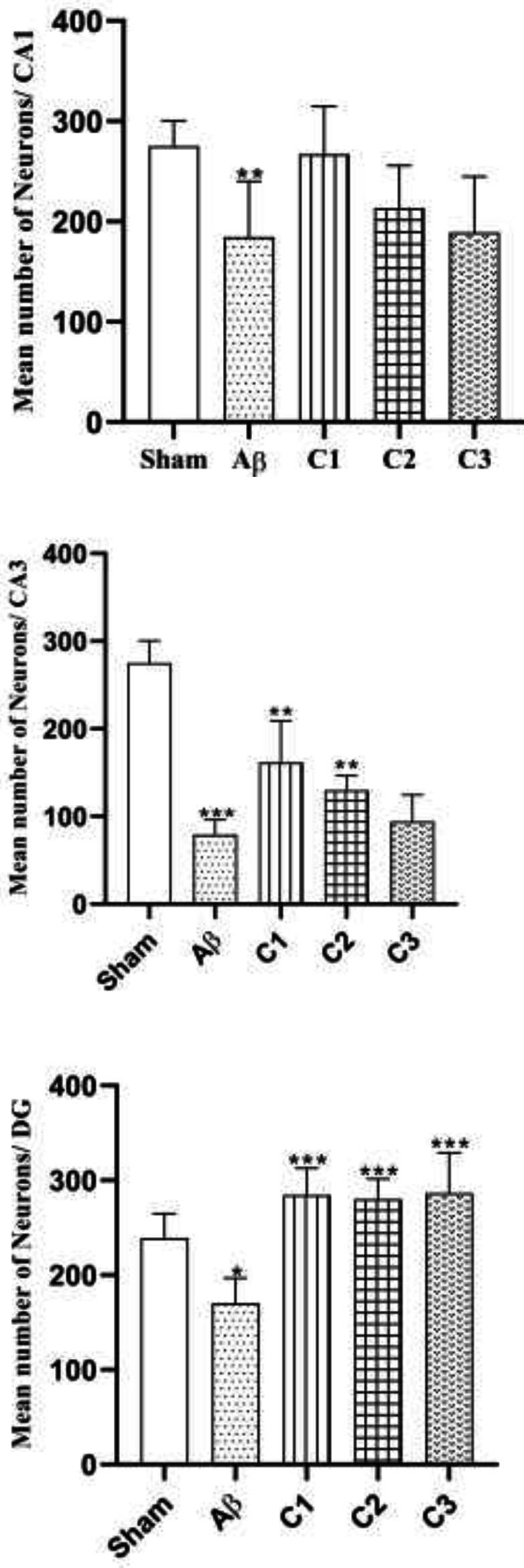
a. The mean number of neurons in CA_1_ in different groups

Figure 12a shows mean number of neurons in all groups. The mean number of neurons in CA1 was 275 ± 8.5 in the sham group, 184.5 ± 18.5 in the lesion group, 267 ± 15.9 in the treated animals with low concentration of silibinin, 213.5 ± 16 in the treated animals with medium concentration of silibinin and 189 ± 18.6 in the animals treated with high concentration of silibinin. 

The mean number of neurons in CA3 of the hippocampus shows in Figure 12b. It was 275 ± 8.5 in the sham group, 79 ± 5.7 in the lesion group, 161.5 ± 15.9 in the low concentration treatment group, 129.8 ± 6.3 in the medium concentration treatment group and 94.11 ± 10.1 in the high concentration treatment group. 

Finally, figure 12c shows the mean number of neurons in the DG. It was 239.2 ± 12.2 in the sham group, 170.5 ±8.9 in the lesion group, 284.5 ±9.4 in the low concentration treatment group, 280.2 ±8 in the medium concentration treatment group and 286.4± 14.2 in the high concentration treatment group. 

**** sham vs. lesion group; *P*= 0.0001, **** C1 *vs*. lesion group; *P*=0.0001, *C2 *vs*. lesion group; *P*=0.05, ***C3 *vs.* lesion group; *P*=0.001

*** sham* vs*. lesion group;* P*=0.0007, ** C_1 _*vs.* lesion group;* P*=0.001

**** sham *vs*. lesion group; *P *=0.001, **** C1 *vs*. lesion group; *P*=0.001, *C1 *vs*. C_2_ group; *P*=0.001, ***C_1_ *vs*. C_3_ group *P*=0.001

*Sham* vs*. lesion group; *P *= 0.03. *C_1_, C_2_, C_3_ *vs*. lesion group; *P*=0.01

(C_1_ = 50 mg / kg, C_2_ = 100 mg / kg, C_3_ = 200mg / kg)

**** sham vs. lesion group; *P*= 0.002, **** C1 *vs*. lesion group; *P*=0.01, $$$ C_2_ *vs*. lesion group; *P*≥ 0.01, &&C_3_ *vs*. lesion group *P*=0.01

** sham *vs*. lesion group; *P*≤0.001, # C_1_, C_2_ *vs*. lesion group; *P*≤0.05, ## C3 *vs*. lesion group *vs*≤0.001

** sham vs. lesion group; *P*=0.001, # C_1_ *vs*. lesion group; *P*≤0.05, # C_2_ *vs*. lesion group; *P*≤ 0.05, # C_3_ *vs*. lesion group *P*≤0.05

ROS: reactive oxygen species

** sham vs. lesion group; *P*≤0.001, ##C_2_ *vs*. lesion group; *P*=0.001, ## C_3_ *vs*. lesion group; *P*=0.001

*Control *vs*. Aβ; *P*=0.001, * C_1 _and C_2_ *vs*. Aβ; *P*= 0.001, **C_3_ *vs*. Aβ; *P*≤0.001

**; Sham vs. Aβ; *P*= 0.0004. b. The mean number of neurons in CA_3_ in different groups. *** Sham *vs*. Aβ; *P*= 0.0002, ** Aβ *vs*. low dose treatment (C_1_); *P*<0.0001. c. The mean number of neurons in DG in different groups. *Sham *vs*. Aβ; *P*<0.01, ***Aβ *vs*. different doses of silibinin (C_1_, C_2_, C_3_); *P*<0.0001

## Discussion

The silibinin treatment of Aβ_1-40_ lesioned rats eased toxin-induced neuronal degeneration and improved learning and memory performance in MWM test and passive avoidance test via amelioration of oxidative stress and neurotrophic factors BDNF and VEGF content.

Unilateral or bilateral injection of Aβ_1-40_ neurotoxin into hippocampus area can be considered as a reliable model of AD in rats, and mice that suits investigation of different cellular, molecular, physiological and other mechanisms involving in the degeneration of pyramidal neurons and to judge the beneficial potency of newly found agents (17, 18). Microinjection of Aβ_1-40_ into hippocampal area; CA1, CA3, or PG finally results in pyramidal neuronal loss, perturbation of synaptic function and decline working memory, long term memory, and spatial memory capacity (19). Similar findings were found in our study, as showed by MWM test in the lesion group. 

Different mechanisms, including mitochondrial diffusion, alterations in membrane permeability, inflammation, synaptic dysfunction, and excitotoxicity (20) have been reported as the main mechanisms underlying Aβ_1-40_ toxicity. 

Oxidative stress enhancement, as the result of an imbalance between formation and the degradation of prooxidant and anti-oxidant defense, is among the crucial factors in neurodegeneration of hippocampal formation in AD and was reported in many published articles before (21). The tissue level of nitric oxide and ROS increased in the lesioned group with Aβ injection, implying oxidative stress development in our rat model. 

Silibinin, the major (70-80%) and most active compound of silymarin (11) exerts neuroprotective, anti-oxidant, anti-cancer and anti-inflammatory effects (12). Moreover, oral administration of Silibinin resulted in decreased ROS and NO content and improved oxidative stress in lesioned treated animals. In the other words, silibinin has attenuated oxidative stress following Aβ_1-40 _injection. To support our findings, Raza *et al*. (2011) showed that the anti-oxidant activity of silymarin is related to the elimination of free radicals by this agent (22). In another investigation on APP / PS1 mice in 2019 by Liang Shen *et al*. administration of silibinin and silymarin could improve the memory impairment and reduce the load of amyloid plaque in mice brains (23) which is in a same direction with our study. Lu and colleagues in 2009 reported that silibinin can improve memory impairment, inflammatory responses, and oxidative stress through nitric oxide synthase (NOS) and tumor necrosis factor α (TNFα) inhibition (24).

The neuroprotective effect of silibinin may be induced via covering the inflammatory cascade in different status (13, 14). Decreasing the levels of inflammatory molecules, TNFα, interleukin-1 beta (IL-1β), and NP are the possible mechanisms found for silibinin stated by Jung *et al*. (25).

BDNF level in brain and blood circulation is reduced in neurodegenerative diseases (26). Abnormal BDNF levels might be because of the chronic inflammatory situation. Neuroinflammation can affect different BDNF-related signaling pathways as well. Gliosis can induce an increase in the level of ROS, which can lead to neurotoxicity that observed in several brain pathologies. Binding of BDNF to TrkB receptor results in different physiological mechanisms. BDNF-dependent phospholipase C-gamma (PLC-γ) might lead to Ca^2+^ increase signaling and inhibition of inflammatory-dependent apoptosis cascade by the inhibition of glycogen synthase kinase 3-beta (GSK-3β). Additionally, BDNF can modulate gene regulation by activating NF-κB that can induce neuronal survival, growth and long-term potentiation (LTP). The role of BDNF in neuroinflammation is strongly related to its ability to induce by NF-κB (26). In our study, Aβ_1-40_ injected animals indicated decreased level of neurotrophic factors VEGF and BDNF, while different concentrations of silibinin increased the content of BDNF and VEGF in the hippocampal tissue of lesioned treated animal. The beneficial effect of silibinin in reducing neurodegeneration of CA1, CA3 and DG can be derived from these important neurotrophic factors since experimental studies show that BDNF and its receptor TrkB and VEGF promote cell survival, positively modulate neuroplasticity and hippocampal neurogenesis (26, 27), and abnormal level of BDNF might be found because of chronic neuroinflammatory state. 

In support of our findings, Song *et al.* showed that silibinin can exert its protective effect in Aβ neurotoxicity through regulating BDNF/TrkB pathway and reducing autophagy in the hippocampus beside increasing BDNF expression and its Tyrosine receptor beside having anti-inflammatory properties (28, 24). Burcu Yön in 2019 reported that silymarin supplementation could improve anxiety, learning and memory-related behavior in diabetic rats by increasing BDNF levels (29).

Furthermore, MTT analysis of hippocampal tissue samples that treated with the different concentrations of silibinin during four weeks showed that silibinin in a dose-dependent manner could increase the growth of hippocampal tissue cells *in vivo*.

## Conclusion

The results of our investigation showed that silibinin has neuroprotective property in the Aβ1-40 rat model of AD through alleviation of oxidative stress via decreasing NO and ROS production and enhancement of the neurotrophic factor BDNF and VEGF. It seems that silibinin may act as a potential candidate to treat AD because of its anti-oxidant and neuroprotective activities.

## Conflicts of Interest

The authors have nothing to declare. 

## References

[1] Mansergh FC, Wride MA, Rancourt DE (2000). Neurons from stem cells: implications for understanding nervous system development and repair. Biochem Cell Biol.

[2] C Qiu, M Kivipelto, E Von Strauss (2009). Epidemiology of Alzheimer’s disease: occurrence, determinants, and strategies toward intervention. Dialogues Clin Neurosci.

[3] Stix G (2010). Alzheimer’s: Forestalling the darkness. Sci Am.

[4] Zhang H, Ma Q, Zhang Yw, Xu H (2012). Proteolytic processing of Alzheimer’s β-amyloid precursor protein. J Neurochem.

[5] Cunnane SC, Plourde M, Pifferi F, Bégin M, Féart C, Barberger-Gateau P (2009). Fish, docosahexaenoic acid and Alzheimer’s disease. Prog Lipid Res.

[6] Schmitt-Schillig S, Schaffer S, Weber C, Eckert G, Muller W (2005). Flavonoids and the aging brain. J Physiol Pharmacol.

[7] Liebl MP, Kaya AM, Tenzer S, Mittenzwei R, Koziollek-Drechsler I, Schild H (2014). Dimerization of visinin-like protein 1 is regulated by oxidative stress and calcium and is a pathological hallmark of amyotrophic lateral sclerosis. Free Radical Bio Med.

[8] Bossy-Wetzel E, Schwarzenbacher R, Lipton SA (2004). Molecular pathways to neurodegeneration. Nat Med.

[9] Purdy P, Ericsson S, Dodson R, Sternes K, Garner D (2004). Effects of the flavonoids, silibinin and catechin, on the motility of extended cooled caprine sperm. Small Ruminant Res.

[10] Schulz V, Hänsel R, Tyler VE (2001). Rational phytotherapy: a physician’s guide to herbal medicine.

[11] Mateen S, Tyagi A, Agarwal C, Singh RP, Agarwal R (2010). Silibinin inhibits human nonsmall cell lung cancer cell growth through cell-cycle arrest by modulating expression and function of key cell-cycle regulators. Mol Carcinog.

[12] Baluchnejadmojarad T, Roghani M, Mafakheri M (2010). Neuroprotective effect of silymarin in 6-hydroxydopamine hemi-parkinsonian rat: involvement of estrogen receptors and oxidative stress. Neurosci Lett.

[13] Geed M, Garabadu D, Ahmad A, Krishnamurthy S (2014). Silibinin pretreatment attenuates biochemical and behavioral changes induced by intrastriatal MPP+ injection in rats. Pharmacol Biochem Be.

[14] Hou Y-C, Liou K-T, Chern C-M, Wang Y-H, Liao J-F, Chang S (2010). Preventive effect of silymarin in cerebral ischemia–reperfusion-induced brain injury in rats possibly through impairing NF-κB and STAT-1 activation. Phytomedicine.

[15] Jangra A, Kasbe P, Pandey SN, Dwivedi S, Gurjar SS, Kwatra M (2015). Hesperidin and silibinin ameliorate aluminum-induced neurotoxicity: modulation of anti-oxidants and inflammatory cytokines level in mice hippocampus. Biol Trace Elem Res.

[16] Paxinos G WC (1998). The Rat Brain in Stereotaxic Coordinates.

[17] Bagheri M, Rezakhani A, Nyström S, Turkina MV, Roghani M, Hammarström P (2013). Amyloid beta1-40-induced astrogliosis and the effect of genistein treatment in rat: a three-dimensional confocal morphometric and proteomic study. PloS One.

[18] Bagheri M, Rezakhani A, Roghani M, Joghataei MT, Mohseni SJJ (2015). Protocol for three-dimensional confocal morphometric analysis of astrocytes. J Vis Exp.

[19] Shankar GM, Walsh DMJMn (2009). Alzheimer’s disease: synaptic dysfunction and Aβ. Mol Neurodegener.

[20] Carrillo-Mora P, Luna R, Colin-Barenque L (2014). Amyloid beta: multiple mechanisms of toxicity and only some protective effects?. Oxid Med Cell Longev.

[21] dos Santos VV, Santos DB, Lach G, Rodrigues ALS, Farina M, De Lima TC (2013). Neuropeptide Y (NPY) prevents depressive-like behavior, spatial memory deficits and oxidative stress following amyloid-β (Aβ1–40) administration in mice. Behav Brain Res.

[22] Raza SS, Khan MM, Ashafaq M, Ahmad A, Khuwaja G, Khan A (2011). Silymarin protects neurons from oxidative stress associated damages in focal cerebral ischemia: a behavioral, biochemical and immunohistological study in Wistar rats. J Neurol Sci.

[23] Shen L, Liu L, Li X-Y, Ji H-F (2019). Regulation of gut microbiota in Alzheimer’s disease mice by silibinin and silymarin and their pharmacological implications. Appl Microbiol Biot.

[24] Lu P, Mamiya T, Lu L, Mouri A, Niwa M, Hiramatsu M (2009). Silibinin attenuates amyloid β25–35 peptide-induced memory impairments: implication of inducible nitric-oxide synthase and tumor necrosis factor-α in mice. J Pharmacol Exp Ther.

[25] Jung UJ, Jeon MT, Choi MS, Kim SR (2014). Silibinin attenuates MPP(+)-induced neurotoxicity in the substantia nigra in vivo. J Med Food.

[26] Lima Giacobbo B, Doorduin J, Klein HC, Dierckx R, Bromberg E, de Vries EFJ (2019). Brain-Derived Neurotrophic Factor in Brain Disorders: Focus on Neuroinflammation. Mol Neurobiol.

[27] Nowacka M, Obuchowicz E (2013). BDNF and VEGF in the pathogenesis of stress-induced affective diseases: an insight from experimental studies. Pharmacol Rep.

[28] Song X, Liu B, Cui L, Zhou B, Liu W, Xu F (2017). Silibinin ameliorates anxiety/depression-like behaviors in amyloid β-treated rats by upregulating BDNF/TrkB pathway and attenuating autophagy in hippocampus. Physiol Behav.

[29] Yön B, Belviranlı M, Okudan NJJoB (2019). The effect of silymarin supplementation on cognitive impairment induced by diabetes in rats. J Basic Clin Physiol Pharmacol.

